# Editorial: Penile cancer in genitourinary oncology

**DOI:** 10.3389/fonc.2022.1040950

**Published:** 2022-09-26

**Authors:** Keini Buosi, Diego Moreira Capibaribe, Luciana SB. Dal Col, Leonardo O. Reis

**Affiliations:** UroScience, School of Medical Sciences, University of Campinas, UNICAMP, and Center of Life Sciences, Pontifical Catholic University of Campinas, PUC-Campinas, Campinas, Brazil

**Keywords:** penile cancer, diagnosis, prognosis, treatment, guideline

The Research Topic *Penile Cancer in Genitourinary Oncology* as part of the Frontiers in Oncology has explored hot topics in this rare disease that needs further development. Authors from Asia, Europe, North and South America have explored important diagnostic, prognostic and therapeutic aspects.

Lack of significant progress in the treatment and management of penile cancer patients in the United States in recent decades is suggested by the increasing trend in incidence-based mortality (IBM) with no significant improvement in the 5-year relative survival rate observed using population-level data from the SEER database [Deng et al.].

A large multicenter Chinese study including 340 penile cancers has identified that phimosis has decreased its prevalence through the years studied while HPV infections rose, becoming a more important etiological factor. HPV types 16 and 18 comprised 88.6% of infections detected and when added type 33, 91% of infections studied are accumulated [Gu et al.].

Other Chinese group sequenced 35 penile cancer patients’ complete exome in order to define a correlation between gene copy number alterations and the disease prognosis. The 5-year survival rate of patients with *MYCN* and *FAK* amplification was 69.2%, and 65.6%, compared to 94.4% and 94.7% in the non-amplification groups, respectively [Yu et al.].

Lymph node status is the most important prognostic factor of penile cancer, and examined lymph node (ELN) count and lymph node density (LND) were independent prognostic factor for overall survival when analyzing 528 patients in the Surveillance, Epidemiology, and End Results cohort from 2010 to 2015 and 156 patients in a Chinese cohort (2006-2016). Using the ROC curve, the recommended cutoff values of ELN and LND were 13 and 9.3%, respectively (P <0.001) [Gao et al.].

Based on the indication for complementary treatment and the already known poor prognosis of extra lymph node extension (ENE) in penile squamous cell carcinomas, a Chinese group studied 234 patients who underwent bilateral inguinal lymph node dissection surgery and has developed a nomogram based on pathological staging and clinical-laboratory data including platelet-lymphocyte ratio and squamous cell carcinoma antigen, capable of predicting the risk of ENE presence [Wu et al.].

Also, deep inguinal lymph node metastasis (ILNM) was the most accurate factor for predicting pelvic lymph node metastasis (PLNM) in a different cohort of 189 Chinese patients. Once involvement of deep ILNs indicates poor prognosis, authors propose that patients with deep ILNM should be staged as pN3 and referred for pelvic lymph node dissection [Yang et al.].

Radioisotope-guided dynamic sentinel node biopsy (DSNB) simultaneously or secondarily after penile surgery has potential to limit morbidity. Among 41 Germany patients, the morbidity rate was 15.8% per inguinal region and intervention was only required in six groins (7.9%). While DSNB is limited by false-negative results, it might be reduced when performed simultaneously to primary tumor resection [Nemitz et al.].

Incipient data has showed therapeutic potential in the use of immune checkpoint inhibitors against penile cancer progressing after chemotherapy, which usually have 06 months median overall survival.

Two patients successfully treated with pembrolizumab are reported, one with high tumor mutational burden and complete response for 38 months and other with PDL-1 expression in penile tumor tissue with partial response for 18 months [Chahoud et al.]. Other two patients with advanced penile squamous cell carcinoma who were administered chemotherapy combined with sintilimab also showed sustained partial and complete response for one and two years, respectively [Mei et al.].

In a European survey, guideline adherence for most treatment recommendations increases with growing annual penile cancer caseload. The probability of a guideline-adherent recommendation increased with each patient treated per year. The type of hospital care (academic vs. non-academic) did not affect guideline adherence in any scenario [Lebentrau et al.].

Brazilian authors brought a compilation on non-coding RNA and its role in penile cancer, as well as the opportunity to use paraffin samples, allowing retrospective studies. Micro RNAs, pi-RNAs, and long non-coding RNAs (LncRNAs) are already known as corelated to disease and are possible tumor biomarkers with potential diagnostic and prognostic targeting in the near future [Pinho et al.]. This research topic selection highlights the current penile cancer landscape, from new technologies with prognostic ability, with the lymph node dissemination still as the Achilles’ heel, to the great therapeutic potential of immunotherapies for the near future.

## Author contributions

LR: Supervision; KB, DC, LD: Manuscript writing/editing. All authors contributed to the article and approved the submitted version.

## Funding

Ministério da Ciência e Tecnologia/Conselho Nacional de Desenvolvimento Científico e Tecnológico - CNPq, processo 304747/2018-1.

## Conflict of interest

The authors declare that the research was conducted in the absence of any commercial or financial relationships that could be construed as a potential conflict of interest.

## Publisher’s note

All claims expressed in this article are solely those of the authors and do not necessarily represent those of their affiliated organizations, or those of the publisher, the editors and the reviewers. Any product that may be evaluated in this article, or claim that may be made by its manufacturer, is not guaranteed or endorsed by the publisher.

